# Variation in Phenolic Compounds, Antioxidant and Antibacterial Activities of Extracts from Different Plant Organs of Meadowsweet (*Filipendula ulmaria* (L.) Maxim.)

**DOI:** 10.3390/molecules28083512

**Published:** 2023-04-16

**Authors:** Tamara Savina, Valery Lisun, Pavel Feduraev, Liubov Skrypnik

**Affiliations:** 1 Higher School of Living Systems, MedBio Cluster, Immanuel Kant Baltic Federal University, Kaliningrad 236040, Russia; tsavina@stud.kantiana.ru (T.S.); vlisun@stud.kantiana.ru (V.L.); pfeduraev@kantiana.ru (P.F.); 2 Laboratory of Natural Antioxidants, Research and Education Center “Industrial Biotechnologies”, MedBio Cluster, Immanuel Kant Baltic Federal University, Kaliningrad 236040, Russia

**Keywords:** wild medicinal plants, secondary metabolites, phytochemicals, polyphenols, salicylic acid, spiraeoside, health benefits

## Abstract

Meadowsweet (*Filipendula ulmaria* (L.) Maxim.) has been widely used in the treatment of various diseases. The pharmacological properties of meadowsweet are derived from the presence of phenolic compounds of a diverse structure in sufficiently large quantities. The purpose of this study was to examine the vertical distribution of individual groups of phenolic compounds (total phenolics, flavonoids, hydroxycinnamic acids, catechins, proanthocyanidins, and tannins) and individual phenolic compounds in meadowsweet and to determine the antioxidant and antibacterial activity of extracts from various meadowsweet organs. It was found that the leaves, flowers, fruits, and roots of meadowsweet are characterized by a high total phenolics content (up to 65 mg g^−1^). A high content of flavonoids was determined in the upper leaves and flowers (117–167 mg g^−1^), with high contents of hydroxycinnamic acids in the upper leaves, flowers, and fruits (6.4–7.8 mg g^–1^); high contents of catechins and proanthocyanidins in the roots (45.1 and 3.4 mg g^–1^, respectively); and high tannin content in the fruits (38.3 mg g^–1^). Analysis of extracts by high-performance liquid chromatography (HPLC) showed that the qualitative and quantitative composition of individual phenolic compounds in various parts of the meadowsweet varied greatly. Among the flavonoids identified in meadowsweet, quercetin derivatives dominate, namely quercetin 3-*O*-rutinoside, quercetin 3-β-d-glucoside, and quercetin 4′-*O*-glucoside. Quercetin 4′-*O*-glucoside (spiraeoside) was found only in the flowers and fruits. Catechin was identified in the leaves and roots of meadowsweet. The distribution of phenolic acids across the plant was also uneven. In the upper leaves, a higher content of chlorogenic acid was determined, and in the lower leaves, a higher content of ellagic acid determined. In flowers and fruits, a higher contents of gallic, caftaric, ellagic, and salicylic acids were noted. Ellagic and salicylic acids were also dominant among phenolic acids in the roots. Based on the results of the analysis of antioxidant activity in terms of the ability to utilize the radicals of 2,2-diphenyl-1-picrylhydrazine (DPPH) and 2,2′-azino-bis(3-ethylbenzthiazolino-6-sulfonic acid) (ABTS) and in terms of iron-reducing ability (FRAP), the upper leaves, flowers, and fruits of meadowsweet can be considered plant raw materials suitable to obtain extracts with high antioxidant activity. Extracts of plant fruits and flowers also showed high antibacterial activity against the bacteria *Bacillus subtilis* and *Pseudomonas aeruginosa.*

## 1. Introduction

Meadowsweet (*Filipendula ulmaria* (L.) Maxim.) is a perennial medicinal plant that has long been used in traditional medicine and has a wide range of pharmacological effects [1]. Meadowsweet is widely distributed in Europe, Asia, the European part of Russia, and the territory of western and eastern Siberia. The plant is found in wetlands, in wet meadows, on slopes, and along the banks of water bodies [2].

Meadowsweet is a large herbaceous plant with a height of up to 100–150 cm, with an erect, smooth-ribbed, thickly deciduous stem and a lobular root system represented by a short creeping rhizome. The stem leaves are regular, located throughout the stem, up to the inflorescence. The flowers are collected in a dense paniculate inflorescence up to 20 cm long. The fruit is dry and 0.3–0.4 mm in length [2,3]. On the territory of northern and eastern Europe, meadowsweet blooms in June–July and bears fruit in July–August.

Herbal preparations and/or meadowsweet flowers have traditionally been used since the late 16th and 17th centuries to treat inflammatory diseases and as a diuretic and antirheumatic agent [4]. In addition, water extracts, tinctures, and ointments based on meadowsweet are used in the treatment of gout, pneumonia, influenza, urinary tract infections, headache, gastrointestinal disorders, and increased acidity and heartburn and as a wound-healing agent [5,6,7]. Meadowsweet extracts have also been shown to exhibit hepatoprotective, anti-inflammatory, cytotoxic, antibacterial, antioxidant, nootropic, anticarcinogenic, and other biological activities [1,8,9].

It is believed that the biological activity and medicinal properties of meadowsweet are primarily due to the presence of phenolic secondary metabolites. It was previously reported that the composition of meadowsweet includes three main classes of phenolic compounds: phenolic acids and their derivatives (gallic acid, ellagic acid, salicylic acid, methyl salicylate, and salicylic aldehyde), flavonoids and flavonoid glycosides (quercetin, kaempferol, apigenin, catechin, epicatechin, rutoside, hyperoside, spiraeoside, quercitrin, and astragalin), and tannins (tellimagrandin I and II and rugosin A, B1, B2, D, E1, and E2) [5,10].

It is known that the distribution of phenolic compounds and other secondary metabolites in the plant is uneven [11]. For example, it has previously been shown that phenolic compounds predominantly accumulate in the reproductive organs of some plants, while the stems are characterized by the minimum content of these secondary metabolites [12,13]. A number of other studies have shown that the maximum content of phenolic compounds is characteristic of plant leaves [14,15]. 

It is worth noting that despite extensive study of the chemical composition and biological activity of meadowsweet plants, most studies have been devoted to the study of only the aerial part of the plant as a whole [4,5,10,16,17,18] or its flowers [2,19,20,21]. Much less research has been associated with the study of the underground part of the meadowsweet plant [5,18]. Furthermore, insufficient attention from researchers has been paid to the fruits of meadowsweet [22]. Due to the fact that the meadowsweet is a massively tall plant (up to 1.5 m) and based on the hypothesis of an uneven distribution of phytochemicals not only in various organs of the plant but also throughout the plant as a whole, the purpose of this work was to study the quantitative content of individual groups and individual phenolic compounds in various organs and parts (leaves and stems of the upper, middle, and lower parts of the plant, as well as the flowers, fruits, and roots) of the meadowsweet plant. Antioxidant and antibacterial activities of extracts from these parts and organs were also investigated.

## 2. Results

### 2.1. Content of Some Groups of Phenolic Compounds in Different Plant Parts of Meadowsweet

The leaves, flowers, and fruits of meadowsweet were found to be characterized by a high total content of phenolic compounds (Table 1). The level of phenolic compounds in these parts ranged from approximately 59.62 to 64.65 mg g^−1^. The roots of meadowsweet were characterized by a medium content of phenolic compounds (53.12 ± 0.60 mg g^−1^). The total content of phenolic compounds in the stems was approximately three times lower compared to that in the leaves.

Among the phenolic compounds in meadowsweet, a high total flavonoid content was noted (Table 1). The maximum content of flavonoids was determined in flowers (about 167 mg g^−1^). Furthermore, a high content of this group of compounds was noted in the leaves, and a trend towards a decrease in the content of flavonoids from the leaves of the upper part of the plant to the leaves of the lower part of the plant (from 117 to 66 mg g^−1^, respectively) was revealed. Low content of flavonoids was established in the stems and roots of the plant (about 10–20 times lower compared to the leaves).

Another important class of phenolic compounds of meadowsweet is hydroxycinnamic acids, the change in the content of which in different parts of the plant was similar to that of flavonoids. The maximum total content of hydroxycinnamic acids was established in the upper leaves of the plant and flowers (Table 1). In addition, the meadowsweet fruits were distinguished by a high content of hydroxycinnamic acids. 

A study of the total content of catechins in various parts of the meadowsweet plant showed that, unlike flavonoids and hydroxycinnamic acids, a high content of catechins was characteristic of the roots of the plant (about 45 mg g^−1^) (Table 1). In addition, an increase in the total content of catechins from the top of the plant to the bottom was found for the leaves and stems.

The maximum content of proanthocyanidins was determined in the leaves of the upper part and the roots of meadowsweet (3.07 ± 0.23 and 3.37 ± 0.24 mg g^−1^, respectively) (Table 1). The content of catechins in the stems and generative parts of the meadowsweet was 4–10 times lower than that in the leaves. 

A study of the distribution of tannins in meadowsweet plant showed that a higher content of tannins was present in the fruits, roots, and leaves of the lower part of the plant than in the upper part of the plant. Moreover, 1.3-fold and 2-fold increases in the content of tannins from the upper parts to the lower parts were established for leaves and stems, respectively.

### 2.2. Content of Individual Phenolic Compounds in Different Plant Parts of Meadowsweet

**Figure A1:**
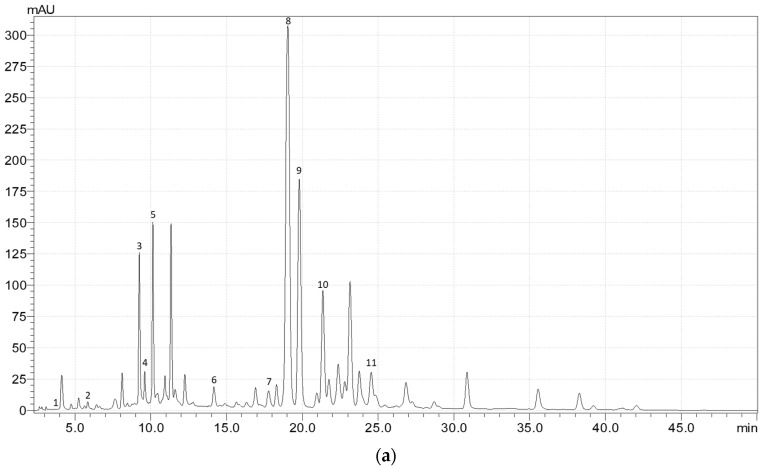

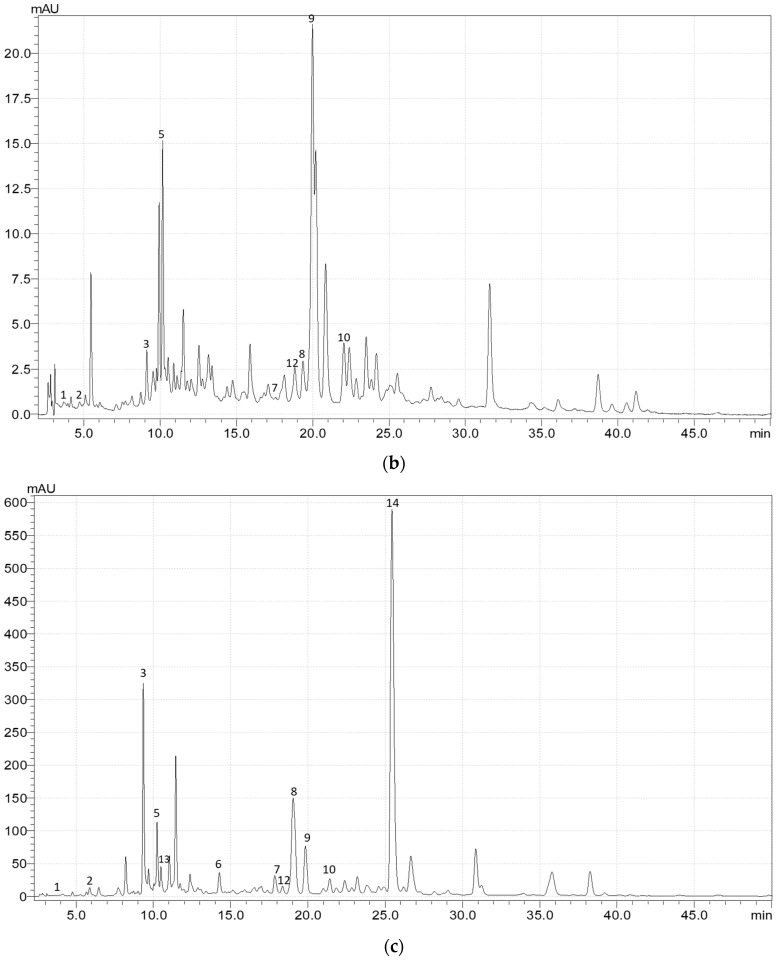

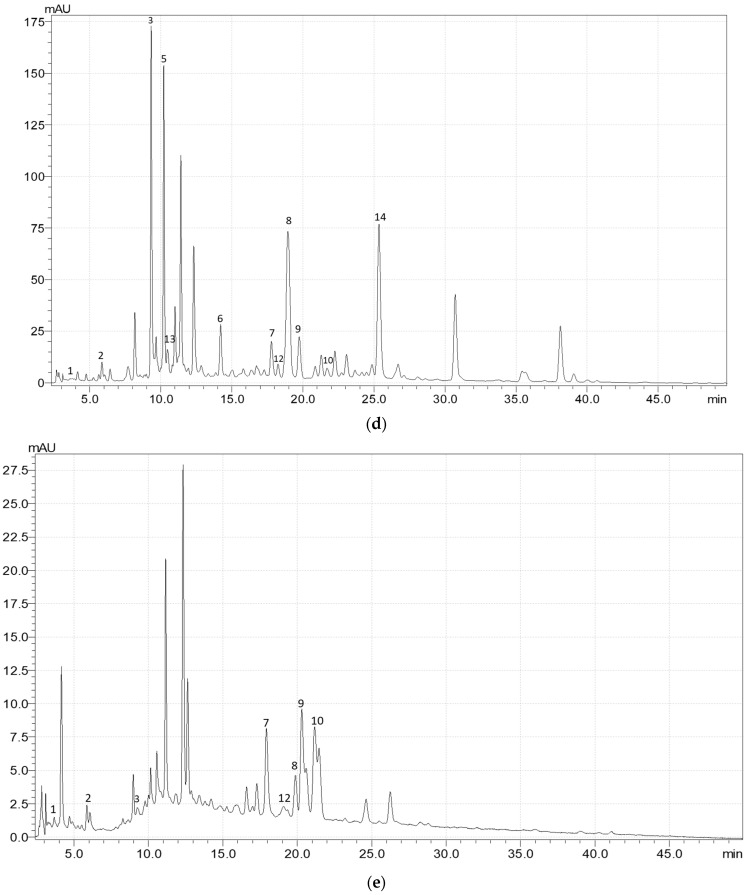
Exemplary HPLC chromatogram (at 340 nm) of the phenolic acids and flavonoids in (**a**) the upper leaves, (**b**) upper stems, (**c**) flowers, (**d**) fruits, and (**e**) roots of meadowsweet (*Filipendula ulmaria* (L.) Maxim.). 1—gallic acid; 2—3,4-dihydroxybenzoic acid; 3—caftaric acid; 4—catechin; 5—chlorogenic acid; 6—p-coumaric acid; 7—ellagic acid; 8—rutin; 9—isoquercitrin; 10—cynaroside; 11—astragalin; 12—salicylic acid; 13—caffeic acid; 14—spiraeoside.

Individual flavonoids and phenolic acids in different parts of the meadowsweet were separated and quantified by high-performance liquid chromatography with a diode array detector (HPLC–DAD) (Table 2, Appendix A, Figure A1). Among the identified flavonoids, flavonol derivatives, namely derivatives of quercetin (quercetin 3-*O*-rutinoside, quercetin 3-β-d-glucoside, and quercetin 4′-*O*-glucoside) dominated in meadowsweet. Quercetin 3-*O*-rutinoside and quercetin 3-β-d-glucoside were found in all parts of the meadowsweet plant. However, their higher content was noted in the leaves, especially the upper part of the plant, as well as the flowers and fruits. Quercetin 4′-*O*-glucoside was found only in the generative parts of the meadowsweet plan, and its content in flowers was 10 times higher than that in fruits, at about 20 mg g^−1^.

Catechin was identified in the leaves and roots of meadowsweet. The maximum content of catechin was determined in the roots, at about 8 mg g^−1^. For individual catechin, as well as for the total content of catechins, a tendency of increased content from the leaves of the upper part of the plant to the lower part was observed (from 1.98 to 6.28 mg g^−1^). 

Various parts of the meadowsweet plant differed in the variety of phenolic acids, and their quantitative distribution across the plant was also uneven. A higher content of chlorogenic acid was determined in the leaves than in other parts of the plant (1.04–1.31 mg g^−1^). The leaves of the lower part of the plants also had a high content of ellagic acid (2.09 ± 0.12 mg g^−1^). The flowers and fruits had higher concentrations of gallic (5.82 ± 0.40 and 4.32 ± 0.29 mg g^−1^, respectively), caftaric (2.92 ± 0.21 and 1.54 ± 0.09 mg g^−1^, respectively), ellagic (5.84 ± 0.34 and 3.44 ± 0.24 mg g^−1^, respectively), and salicylic (4.51 ± 0.32 and 1.94 ± 0.08 mg g^−1^, respectively) acids compared to other parts of the meadowsweet. Ellagic and salicylic acids were also dominant among phenolic acids in the roots, although their content was 3–7 times lower compared to the generative parts. It should be noted that despite the fact that the stems were generally characterized by a lower content of individual groups of phenolic compounds, their average content of gallic acid was slightly higher than that in the leaves. Additionally, the stems, especially in the upper part of the plant, were characterized by the accumulation of salicylic acid, the content of which reached about 1 mg g^−1^.

### 2.3. Antioxidant Activity of Extracts from Different Plant Parts of Meadowsweet

The antioxidant activity of extracts was measured by three assays, namely by reaction with 2,2-diphenyl-1-picrylhydrazyl radical (DPPH), by reaction with the radical 2,2′-azino-bis(3-ethylbenzthiazolino-6-sulfonic acid) (ABTS), and by the ability to reduce Fe (III) in complex with 2,4,6-tripyridyl-s-triazine (FRAP). The maximum antioxidant activity measured by all three assays (DPPH, ABTS, and FRAP) was distinguished by extracts from meadowsweet flowers (Table 3). The fruit extracts were also characterized by high antioxidant activity. The antioxidant activity of leaf extracts was 1.5 to 3.0 times lower than that of flowers. However, a higher level of antioxidant activity was found for extracts from the upper leaves, except for antioxidant activity, as measured by the ability to reduce iron (III) (FRAP). In the latter case, extracts from the leaves of the lower part of the plant were also characterized by high antioxidant activity.

### 2.4. Antibacterial Activity of Extracts from Different Plant Parts of Meadowsweet

The antibacterial activity of extracts from various parts of meadowsweet was studied against species of Gram-negative bacteria *Escherichia coli* and *Pseudomonas aeruginosa* and against Gram-positive bacteria *Bacillus subtilis*. It should be noted that in relation to *E. coli*, the antibacterial effect of meadowsweet extracts was not established, whereas in relation to *P. aeruginosa*, the antibacterial an effect was shown for the extracts from flowers, fruits, leaves, roots, and even the stems of the lower part of the plant (Table 4). In relation to *B. subtilis*, antibacterial action was established only for extracts of flowers and fruits.

### 2.5. Correlation and Cluster Analysis Based on the Content of Phenolic Compounds and Antioxidant Activity of Their Extracts

**Figure 1:**
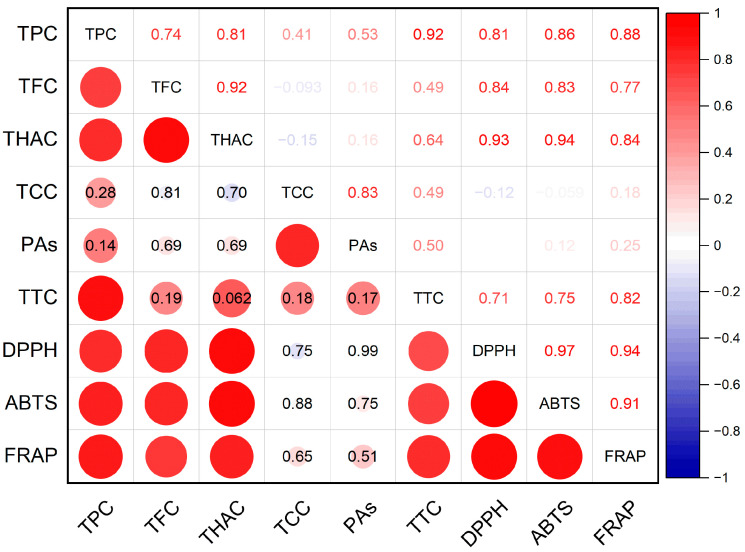
Pearson correlation coefficients between phenolic compounds and antioxidant activity of extracts. TPC—total phenolic content; TFC—total flavonoid content; THAC—total hydroxycinnamic acid content; TCC—total catechin content; PAs—total proanthocyanidin content; TTC—total tannin content; DPPH—antioxidant activity determined by DPPH (2,2-diphenyl-1-picrylhydrazyl) assay; ABTS—antioxidant activity determined by ABTS (2,2′-azino-bis(3-ethylbenzothiazoline-6-sulfonic acid)) assay; FRAP—ferric-reducing antioxidant power.

According to the results of the correlation analysis, a connection was established between the accumulation of hydroxycinnamic acids, flavonoids, total phenolics, and tannins in meadowsweet and the antioxidant activity of extracts (Figure 1). The correlation coefficients between these parameters were 0.71–0.94 (*p* ≤ 0.05). A significant correlation was also found between the accumulation of flavonoids and hydroxycinnamic acids (r = 0.92, *p* ≤ 0.05); between the total contents of phenolic compounds, flavonoids, hydroxycinnamic acids, and tannins (r = 0.74–0.92, *p* ≤ 0.05); and between the contents of catechins and proanthocyanidins (r = 0.83, *p* ≤ 0.05).

The results of antioxidant activity determination of the extracts by the three assays (DPPH, ABTS, and FRAP) correlated well with each other, as reflected by the high values of the correlation coefficients between antioxidant activity parameters (r = 0.91–0.97, *p* ≤ 0.05).

**Figure 2:**
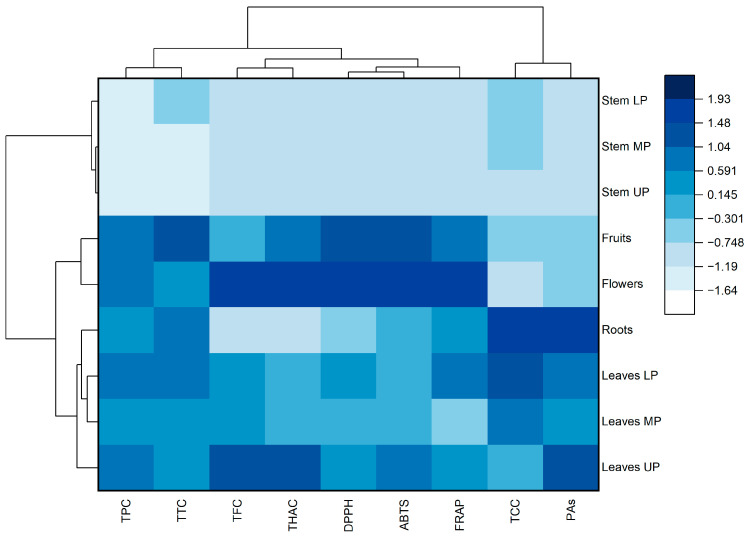
Heat map with clusters of the studied phytochemical parameters (at the **top**) and meadowsweet samples (on the **left**). TPC—total phenolic content; TFC—total flavonoid content; THAC—total hydroxycinnamic acid content; TCC—total catechin content; PAs—total proanthocyanidin content; TTC—total tannin content; DPPH—antioxidant activity determined by DPPH (2,2-diphenyl-1-picrylhydrazyl) assay; ABTS—antioxidant activity determined by ABTS (2,2′-azino-bis(3-ethylbenzothiazoline-6-sulfonic acid)) assay; FRAP—ferric-reducing antioxidant power; LP, MP, UP—lower, middle, and upper parts of plants, respectively.

Based on the normalized values of the studied indicators, a heat map with cluster analysis was generated (Figure 2). The dendrogram shown in the Figure 2 (at the top) demonstrates that all the studied parameters can be divided into two main clusters. The first cluster includes total phenolic compounds, tannins, flavonoids, hydroxycinnamic acids, and antioxidant activity (DPPH, ABTS, and FRAP). The second cluster includes catechins and proanthocyanidins.

Meadowsweet samples were divided into two clusters (Figure 2, left). The first cluster includes stems characterized by low content of most groups of studied phenolic compounds and antioxidant activity of extracts. The second cluster includes the leaves, roots, and generative parts of the meadowsweet plant. Flowers and fruits form a separate subgroup within this cluster.

## 3. Discussion

### 3.1. Meadowsweet as a Natural Source of Phenolic Compounds

In folk medicine, all parts of meadowsweet (flowers, leaves, and roots) have been used for more than four centuries in the treatment of many diseases. Modern studies conducted both in vivo and in vitro prove the anti-inflammatory, antibacterial, anticancer, antidepressant, and antioxidant properties of meadowsweet [1,3,5,16,22,23]. The biological activity of meadowsweet is due to the presence of a number of biologically active components; however, it is of particular importance from a pharmacological point of view as a source of natural phenolic compounds. As a result of this study, it was found that the leaves, flowers, fruits, and roots of the meadowsweet plant are characterized by a high total content of phenolic compounds (up to 65 mg g^−1^), although about two times lower than the previously determined value in meadowsweet grass (about 120 mg g^−1^) presented in a study by Harbourne et al. [17]. Additionally, the flavonoid content (maximum content in flowers was about 170 mg g^−1^) of the samples analyzed in the present study was about 1.6 times higher than the results obtained for meadowsweet flowers in the work of Baranenko et al. [24]. The differences in the results are probably due to the different growing conditions of the plants, as well as the different extraction methods used in the studies. In a study by Harbourne et al., the total content of phenolic compounds was determined in the extracts of aerial parts (a mixture of flowers, stems, and leaves) of meadowsweet collected in Ireland. The extract was obtained using heated distilled water (100 °C) stirred on hot plate [17]. Baranenko et al. investigated the extracts of flowers obtained using ethyl alcohol with a volume fraction of 70%. The plant material was collected from different regions of Russia (Leningrad and Yaroslavl regions, Republic of Bashkortostan) [24]. The variation in the content of phenolic compounds and flavonoids in extracts of meadowsweet in our and referenced studies may be associated with climatic conditions of plant growth, for example, the average air temperature, humidity, and soil composition. These factors are known to significantly affect the content of phenolic compounds in wild plants [25]. However, since none of the discussed studies provided data on the climatic conditions of meadowsweet growth, an accurate interpretation of the results requires additional studies, for example, aimed at investigation of the variation in the content of phenolic compounds in meadowsweet depending on growing conditions.

The results of HPLC–DAD analysis showed that the main flavonoids of meadowsweet are derivatives of quercetin. Additionally, in some samples, derivatives of kaempferol and luteolin were detected. The results obtained in the present study are consistent with the previously obtained data on the composition of phenolic compounds in meadowsweet [20,26]. HPLC–DAD analysis revealed that the distinctive feature of the flowers and fruits of meadowsweet is the presence of spiraeoside (quercetin 4′-*O*-glucoside). This quercetin derivative was not detected in any other parts of the plant. The obtained result is consistent with the data available in the literature, according to which the meadowsweet flowers, along with the outer scales of *Allium cepa*, are the main natural sources of spiraeoside [27,28]. 

Among the phenolic acids in the meadowsweet samples, especially in the generative parts, gallic, ellagic, caftaric, and salicylic acids prevailed. The high content of salicylic acid and its derivatives is also a feature of meadowsweet [4]. It is believed that these phytocomponents cause its anti-inflammatory properties [6]. 

The roots of the meadowsweet plant were characterized by a higher total content of catechins (up to 45 mg g^−1^) and proanthocyanidins (up to 3.4 mg g^−1^) compared to other studied parts. In addition to high antioxidant activity [29], it is believed that catechins can also exert anti-inflammatory effects by regulating iNOS and COX-2 expression and suppressing inflammatory cytokines [30] and antibacterial effects [31]. Katanić et al. found a wide variety of dimers, trimers, tetramers, and galloylated procyanidins in both the roots and extracts of the aerial part of the meadowsweet plant [32]. These proanthocyanidins predominantly consisted of catechin and epicatechin subunits. Proanthocyanidins (also called condensed tannins) are also phytocomponents with potent biological activity, including anticarcinogenic, antimicrobial, antiviral, anti-inflammatory, antiallergic, antimutagenic, and antihyperglycemic effects [33].

A high total content of tannins was determined in meadowsweet fruits (about 40 mg g^−1^), as well as in the leaves—especially in the lower leaves, flowers, and roots. The results obtained in the present study are consistent with the previously reported data, according to which meadowsweet is characterized by a significant content of ellagitannins, primarily tellimagrandins I and II and rugosin D [34]. These hydrolyzable tannins have been found to exhibit anti-inflammatory, anticancer, antioxidant, and antimicrobial (antibacterial, antifungal, and antiviral) activity [35].

The antioxidant activity of plant extracts is due to the presence of various phytocomponents. However, it is believed that the antioxidant potential of plants is mainly due to the qualitative and quantitative composition of phenolic compounds [36], which is consistent with the results of the present study. A close correlation was established between the antioxidant activity of meadowsweet extracts and the contents of hydroxycinnamic acids, flavonoids, total phenolics, and tannins. The most promising method to obtain extracts with high antioxidant activity is the use of the flowers and fruits of meadowsweet, which are characterized by maximum antioxidant activity according to the results of analyses using all three assays (DPPH, ABTS, and FRAP). It is worth noting that data on the antioxidant activity of extracts from meadowsweet fruits were obtained for the first time in this study. The underground part of the meadowsweet plant is also understudied. Only one previous study examining the antioxidant properties of meadowsweet root extracts has been published to date, in which Katanić et al. (2015) compared the antioxidant activity of the extracts of the aboveground and underground parts of meadowsweet. The authors showed that the extracts of the underground part exhibited more antioxidant activity against the radicals DPPH and ABTS [18]. In the present study, root extracts exhibited moderate-strength antioxidant activity. The differences in the results are probably due to the fact that in the work cited above, the authors used the entire aerial part for extraction, including the stem of the plant, which, as shown by the present studies, was characterized by very low antioxidant activity.

Antibiotic resistance is a serious global health problem and a threat to public health. Flavonoids and other phenolic compounds have been actively studied as possible antibacterial agents over the past few years [37]. In the present study, a pronounced antibacterial effect of flower and fruit extracts on Gram-negative bacteria *P. aeruginosa* and Gram-positive bacteria *B. subtilis* was established. Furthermore, with respect to *P. aeruginosa*, the antibacterial effect was also shown by extracts of leaves (especially from the upper part of the plant), roots, and stems from the lower part of the plant. A stronger antibacterial effect of meadowsweet flowers and fruits may be associated with a higher content of the flavonoid spiraeoside (quercetin 4′-*O*-glucoside) and phenolic acids, in particular gallic, ellagic, and salicylic acids. Previously, a number of studies have shown that these phenolic acids and some quercetin derivatives have an antibacterial effect [38,39,40]. With regard to *E. coli*, none of the extracts investigated in the present study showed antibacterial activity. There are conflicting data in the literature on the antibacterial activity of meadowsweet extracts against *E. coli*. The antibacterial effect of extracts of the aboveground and underground parts of meadowsweet against *P. aeruginosa* and *E. coli* was established in a study by Katanić et al. [18], whereas in another study by Papastavropoulou et al. [41], meadowsweet extracts did not show antibacterial activity against *E. coli*. Differences in the obtained results may be associated both with the use of different strains of microorganisms for research and with the difference in plant material and the preparation of extracts. In contrast to our study and the study by Papastavropoulou et al. [41], in which aqueous solutions of ethanol or methanol were used for extraction, in the work of Katanić et al. [18], extraction was carried out with pure methanol as the solvent. The use of methanol generally results in a higher content of the more lipophilic non-glycosylated forms of phenolic compounds and polymeric polyphenols. In a study by Metrouh-Amir et al., it was also shown that the extract of *Matricaria pubescens* prepared with pure methanol had a higher antibacterial effect against *E. coli* (KB 349) compared to the aqueous methanol extract [42].

### 3.2. Vertical Distribution of Phenolic Compounds in Meadowsweet Plant

To describe the nature of the accumulation and distribution of phenolic compounds in various organs of meadowsweet plants, a fairly wide range of factors must be taken into account [43,44,45]. This is, on the one hand, a set of exogenous environmental factors that take into account the specifics of growth and, on the other hand, a set of endogenous factors, such as the species specificity of the accumulation of phenolic secondary compounds and the growing season during which plant material is collected. Each of these factors or their combination can significantly affect the production of this group of secondary metabolites [46,47]. In present study, the collection of plant material was carried from a homogeneous phytocenosis during the period of mass flowering and fruiting. This period was chosen as it is characterized by the maximum concentration of phenolic compounds in many species of higher plants, such as plants of the genus *Rumex*, sweet marjoram (*Origanum majorana* L.), *Opuntia*, and *Withania somnifera* (L.) Dunal [13,48,49,50]. The observed accumulation of phenolic compounds in plants in the flowering stage may be associated with an increase in the activity of the key enzyme for the synthesis of phenolic compounds such as phenylalanine ammonia-lyase (PAL) and, probably, with an increase in the activity of other enzymes of metabolic pathways of flavonoid biosynthesis and transformations. In previous studies, it was found that the concentration of PAL in plants reached a maximum at the time of initiation of flowering and mass flowering [51,52].

However, peak accumulation values in the whole plant do not provide sufficiently complete information on the distribution of phenolic compounds in various vegetative and generative organs of the plant. The search for characteristic patterns of deposition of phenolic compounds in meadowsweet plants is complicated by the scarcity and fragmentation of experimental research data within this object. Generally, the distribution of secondary metabolites, including phenolic compounds, can be considered from several perspectives: (i) localized organospecific/tissue-specific synthesis, (ii) transport of “early” precursors of the synthesis of phenolic compounds (sugar), and (iii) transport of “late” precursors of synthesis (in the case of phenolic compounds, “late” precursors are compounds with an aromatic group) [53]. The central tissue compartment for the biosynthesis of phenolic compounds is mesophyll, which is predominantly concentrated in the leaves. However, the age of the leaf plate can largely determine the metabolic profile of phenolic compounds. Therefore, in young leaves located closer to the inflorescences, the presence of redox-active flavonoids and hydroxycinnamic acids was noted. Conversely, in older leaves, there an increased content of compounds with a potency for oligomerization/polymerization (catechins and tannins) was observed. Although the data regarding the effect of the age of leaves on metabolic productivity are somewhat inconsistent, most authors lean toward the hypothesis that young tissues generally have higher rates of synthetic activity [54,55,56]. The results of antioxidant activity tests indirectly confirm this hypothesis. The redox status of older leaves (associated with lower internodes) is significantly lower compared to that of younger leaves [57].

The main contribution to the formation of the phenolic profile of the stem part of the studied species is photosynthetic mesophilic tissue and/or the metabolic profile of the phloem exudate. Since the proportion of mesophyll in the stem tissue is not high, especially in comparison with mechanical tissues that form a rigid vertical structure of the aboveground part of the plant, the transport of phenolic compounds and/or their precursors is the main factor determining the phenolic profile of stems. However, it should be noted that phenolic compounds are not typical compounds for phloem transport. However, some authors point to the presence of small amounts of phenolic substances in the phloem exudate [58,59]. One of the factors preventing the free flow of phenols through the channels of the phloem is the alkaline reaction of the solution of the central cavity, in which these compounds are especially sensitive to oxidation, forming aggressive quinones [60]. The low representation of metabolically active tissues and a redox-inactive phloem exudate characterize the stem as an anatomical structure that does not accumulate and does not store biologically active compounds in large quantities.

The biosynthesis of secondary metabolites in the roots and generative parts of the meadowsweet plant is mainly associated with the transportation of photoassimilates. The most characteristic components of phloem exudate are carbohydrates, which are the most typical precursors in the biosynthesis of phenolic metabolites [61,62]. The difference in accumulation strategies and expression activity of genes encoding the enzymes involved in the formation of phenolic compounds in the tissues of the generative parts and tissues of the root system determine the final composition of phenolic compounds in these organs. The results of the present study demonstrate a high content of flavonoids and phenolic acids in the flowers and a high content of catechins and proanthocyanidins in the roots of meadowsweet. The latter two compounds are quite easily involved in oligomerization reactions, and, in fact, are elements of the “dead-end” branch of flavonoid metabolism and actively stored in tissues [63].

## 4. Materials and Methods

### 4.1. Plant Samples

**Figure 3:**
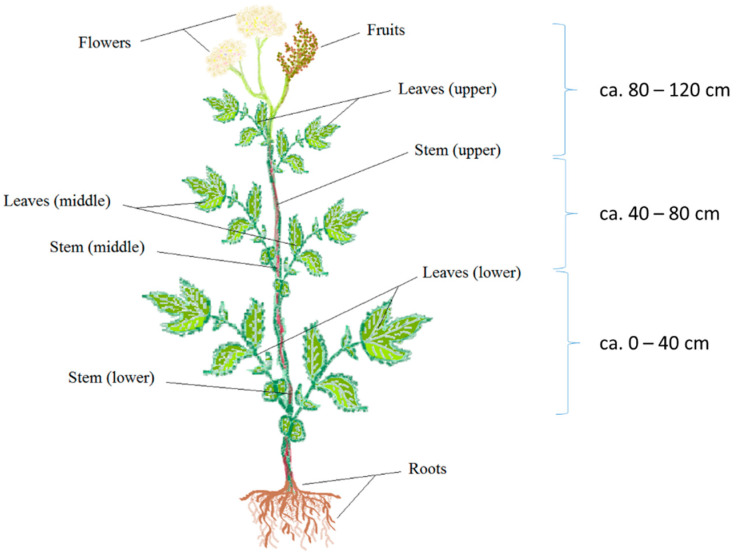
Scheme of the analysis of the vertical distribution of phenolic compounds in meadowsweet plants.

Meadowsweet (*Filipendula ulmaria* (L.) Maxim.) was collected in July 2020 in a place of natural growth in the Kaliningrad region (altitude, 29 m above sea level; longitude 20°26′ E; latitude, 54°45′ N). Plant harvesting was carried out during the stages of mass flowering and the beginning of fruiting from four test sites with a size of 50 square meters. From the test site, 5–6 plants were selected. The collected plants were divided into 9, as shown in Figure 3. The same parts and organs of all collected plants from the same site made up the combined sample. Vegetable material from each test site was analyzed separately (*n* = 4).

The plant material was dried at 40 °C to a constant weight and crushed to the size of particles passing through a sieve with a hole diameter of 2 mm.

### 4.2. Preparation of Extracts

The phenolic compounds were extracted from the ground dry plant material with a 60% aqueous ethanol solution. A 0.5 g plant material sample was placed in a round-bottom flask spiked with approximately 50 mL of 60% ethanol and heated at 45 °C in a reflux water bath for 1 h. Ethanol concentration, the solvent-to-solid ratio, and extraction time and temperature were selected in preliminary experiments to optimize extraction conditions using the response surface methodology. The mixture was then filtered into a volumetric flask. The extraction procedure was repeated three times. The resulting portions of the extract were mixed and adjusted to 100 mL with 60% ethanol.

The resulting extract was used to determine the total contents of phenolic compounds, flavonoids, hydroxycinnamic acids, catechins, proanthocyanidins, and tannins, as well as antioxidant and antibacterial activities, and to analyze individual phenolic compounds using HPLC–DAD. Prior to chromatographic analysis, the extract was further filtered through a syringe filter (0.22 µm). To study antibacterial activity, the extract was evaporated on a rotary evaporator and dissolved in 8% aqueous dimethyl sulfoxide (DMSO) prior to analysis.

### 4.3. Determination of Phenolic Compounds

The total contents of phenolic compounds were determined with Folin–Ciocalteu reagent [64]. Briefly, 2.5 mL of plant extract obtained as described above or standard solution was mixed with 1.25 mL 0.2 M Folin–Ciocalteu reagent, placed in darkness, and incubated for 10 min at room temperature. Then, 2.5 mL of 7.5% sodium carbonate solution was added to the mixture, and the reaction mixture was incubated for 30 min in darkness at room temperature. The absorbance of the solutions was measured at 765 nm using a UV-3600 spectrophotometer (Shimadzu, Kyoto, Japan). Gallic acid was used as the standard. The total content of phenolic compounds was expressed in mg of gallic acid equivalents per gram of dry mass of the plant (mg GAE g^−1^). All measurements were carried out in triplicate.

The total content of flavonoids was determined by the reaction of complexation with AlCl_3_ in the presence of 1 M potassium acetate [65]. The reaction mixture consisted of 500 μL extract or standard solution, 1.5 mL of 95% ethanol, 100 μL of 10% (*m*/*v*) aluminum chloride, 100 μL of 1 M potassium acetate, and 2.8 mL of distilled water. The mixture was vortexed, then incubated at room temperature for 30 min. The absorbance of the solutions was measured at 415 nm using a UV-3600 spectrophotometer (Shimadzu, Kyoto, Japan). Rutin was used as the standard. The total flavonoid content was expressed in mg of rutin equivalents per gram of dry weight of the plant (mg RE g^−1^). All measurements were carried out in triplicate.

The total content of hydroxycinnamic acids was determined using Arnow reagent according to the protocol previously described in the European Pharmacopoeia [66]. Test solution was prepared by mixing 1.0 mL of extract or standard solution, 2 mL of 0.5 M hydrochloric acid, 2 mL of Arnow reagent (prepared by dissolving 10 g of sodium nitrite and 10 g of sodium molybdate in 100 mL of water), 2 mL of sodium hydroxide solution (8.5%, *m*/*v*), and 3 mL of distilled water. The absorbance of the solutions was measured at 525 nm using a UV-3600 spectrophotometer (Shimadzu, Kyoto, Japan). Chlorogenic acid was used as the standard. The total hydroxycinnamic acid content was expressed in mg of chlorogenic acid equivalents per gram of dry plant mass (mg CAE g^−1^). All measurements were carried out in triplicate.

The total content of catechins was determined spectrophotometrically using vanillin reagent according to [67] with some modifications. Briefly, 1 mL of plant extract or standard solution was mixed with 4 mL of vanillin reagent (1% solution of vanillin in concentrated HCl). A mixture of plant extract (or standard) and concentrated HCl (without vanillin) was used as the blank solution. The reaction mixture was incubated for 5 min at room temperature. The absorbance of the solutions was measured at 520 nm using a UV-3600 spectrophotometer (Shimadzu, Kyoto, Japan). Catechin standard solutions were used to generate the calibration plot. The total content of catechins was expressed in mg of catechin equivalents per gram of dry mass of the plant (mg CE g^−1^). All measurements were carried out in triplicate.

The total content of proanthocyanidins was determined using a butanol–salt reagent with the addition of ferric ammonium sulfate as a catalyst according to [68]. The reaction mixture consisted of 9 mL of acidified butanol containing iron sulfate (77 mg FeSO_4_ × 7H_2_O in 500 mL HCl/BuOH (2/3)) and 1 mL of plant extract. The reaction mixture was incubated in a water bath at 95 °C for 30 min. The absorbance of the solutions was measured at 520 nm using a UV-3600 spectrophotometer (Shimadzu, Kyoto, Japan). The total content of proanthocyanidins was expressed in mg of cyanidin equivalents per gram of dry weight of the plant (mg CyE g^−1^). All measurements were carried out in triplicate.

The total content of tannins was determined spectrophotometrically by the reaction of the formation of Prussian blue [12]. During the first stage of the analysis, the total content of polyphenols was determined using iron (III) chloride and potassium ferricyanide. Briefly, 250 μL of the extract or standard solution was mixed with 25 mL of distilled water, and 3 mL of a 0.5 M solution of FeCl_3_ and 3 mL of 0.008 M K_3_Fe(CN)_6_ were added. The absorbance of the solutions was measured at 720 nm after incubation for 15 min using a UV-3600 spectrophotometer (Shimadzu, Kyoto, Japan). The tannins were then precipitated from the extract by casein as described in [69]. Briefly, 0.24 g of casein was added to 10 mL of extract, and the mixture was stirred and incubated at 30 °C for 1 h. The resulting mixture was filtered, and the content of polyphenols was determined in the filtrate as described above. The difference between the results before and after the deposition of tannins was taken as the content of tannins. Gallic acid was used to generate the calibration plot. The total content of tannins was expressed in mg of gallic acid equivalents per gram of dry mass of the plant (mg GAE g^−1^ DW). All measurements were carried out in triplicate.

Determination of individual phenolic compounds was performed using HPLC–DAD (Shimadzu LC-20 Prominence chromatograph, Shimadzu, Kyoto, Japan). A Phenomenex Luna column (C18 250 × 4.6 mm, 5 µm; Phenomenex, Torrance, CA, USA) was used for the chromatographic separation of the compounds. Elution conditions: eluent: a mixture of water/trifluoroacetic acid (*v*/*v*) 99.9/0.1 (A) and acetonitrile (B); elution mode: gradient elution (0 min: 95% A and 5% B; 3 min: 88% A and 12% B; 46 min: 75% A and 25% B; 49.5 min: 10% A and 90% B; 52 min: 10% A and 90% B; 52.7 min: 95% A and 5% B; 59 min: 95% A and 5% B); flow rate: 0.85 mL/min; sample volume: 20 μL; column temperature: 40 °C. Detection was carried out in the wavelength range of 180–750 nm. The compounds were identified by comparing the retention times of the peaks and UV spectra obtained in the chromatograms with the corresponding parameters of the standards of chromatographically pure samples. The chromatograms were processed using LabSolutions (LC Solution version 1.25SP1 Single PDA) software (Shimadzu, Kyoto, Japan). Quantitative analyses of the phenolic compounds were performed using calibration plots constructed in a concentration range of 10–100 µg/mL. The following standards were used in the study: 3,4-dihydroxybenzoic acid (protocatechuic acid), caftaric acid, chicoric acid, chlorogenic acid, *p*-coumaric acid, trans-caffeic acid, ellagic acid, gallic acid, rosmarinic acid, salicylic acid, luteolin 7-*O*-glucoside, apigenin 7-*O*-glucoside, apigenin 7-*O*-glucuronide, quercetin 3-*O*-rutinoside (rutin), quercetin 3-β-d-glucoside (isoquercitrin), quercetin 3-*O*-galactoside (hyperoside), quercetin 4′-*O*-glucoside (spiraeoside), kaempferol 3-*O*-glucoside (astragalin), baicalin, diosmin, and catechin. All standards were purchased from Sigma-Aldrich (Sigma-Aldrich Rus, Moscow, Russia).

### 4.4. Determination of Antioxidant Activity

The antioxidant activity (AOA) of the extracts was determined by three methods, namely by the reaction with 2,2-diphenyl-1-picrylhydrazyl radical (DPPH), by the reaction with the radical 2,2′-azino-bis(3-ethylbenzthiazolino-6-sulfonic acid) (ABTS), and by the ability to reduce Fe (III) in complex with 2,4,6-tripyridyl-s-triazine (FRAP) [70]. 

For determination of AOA by DPPH assay, 30–100 μL of plant extract was mixed with 2.85 mL of a prepared 0.1 mM solution of DPPH radical in 96% EtOH. The sample was incubated for 30 min at room temperature in darkness. The reduction in absorbance at 515 nm was measured spectrophotometrically.

For the ABTS assay, 2.85 mL of ABTS solution was mixed with 150 μL of plant extracts. ABTS radical was generated by mixing aliquot parts of 7.0 mM ABTS solution and 2.45 mM potassium persulfate solution. After exactly 15 min, the absorbance of the reaction mixture was measured at 734 nm.

In the FRAP-assay, the reaction was started by mixing 3.0 mL of FRAP reagent with 100 μL of plant extract. The FRAP reagent was freshly prepared by mixing 10 parts of 0.3 M acetate buffer (pH 3.6), 1 part of 10 mM 2,4,6-tripyridyl-triazine (TPTZ) in 40 mM HCl, and 1 part of 20 mM FeCl_3_ × 6H_2_O. After 10 min of incubation at 37 °C in darkness, the absorbance was measured at 593 nm.

The absorbance in all assays was measured using a UV-3600 spectrophotometer (Shimadzu, Kyoto, Japan). A mixture containing the appropriate reagent and 60% ethanol was used as a blank solution in the DPPH, ABTS, and FRAP assays instead of extract. Ascorbic acid was used as the standard in all assays. The assay results were expressed in mg of ascorbic acid equivalents per gram of dry weight of the plant (mg AsA g^−1^). All measurements were carried out in triplicate.

### 4.5. Determination of Antibacterial Activity

Three types of bacteria were used to test the antibacterial activity of plant extracts, namely Gram-positive *Bacillus subtilis* (ATCC 6633) and Gram-negative *Escherichia coli* (ATCC 25922) and *Pseudomonas aeruginosa* (ATCC 27853). A single colony of the test organism was taken for planting in a liquid culture. The culture process lasted 12 h to obtain a liquid suspension culture with a turbidity of up to 0.5 according to the McFarland standard (approximately 1–2 × 10^8^ colony-forming units (CFU) per mL). 

The antibacterial activity of plant extracts was assessed by disk diffusion analysis [71]. Inoculation on LB solid medium was performed using sterile cotton buds immersed in suspensions of test microorganisms. Various concentrations of the extract (10, 20, 30, and 40 mg mL^−1^) were applied to the paper disk to obtain a final concentrations of 0.1, 0.2, 0.3, and 0.4 mg of extract per disk, respectively. A kanamycin antibiotic disk (25 µg per disk) was selected as the positive control, and 10 µL of 8% DMSO solution was used as the negative control. Antibacterial activity was determined by measuring the inhibition zone around the disk in millimeters.

### 4.6. Statistical Analysisd

Statistical processing of the study results was carried out using the OriginPro 2022 program (OriginLab Corporation, Northampton, MA, USA). The obtained data are presented as mean ± standard deviation (*n* = 4). Statistical analysis of differences in phenolic compounds and antioxidant activity between different parts of plants was carried out using a one-way analysis of variance, followed by assessment of differences between groups using the Tukey test. The Pearson correlation coefficient was used to evaluate the correlation between the studied biochemical parameters. Differences in means and correlation were considered statistically significant at *p* ≤ 0.05. The heat map and clusters are based on the normalized values of the studied parameters. The Euclidean distance was used as a measure of similarity.

## 5. Conclusions

Studies on the variability of the qualitative and quantitative composition of phenolic compounds in different parts of the meadowsweet showed the heterogeneity of their distribution in the plant. The highest contents of flavonoids and phenolic acids were found in the upper leaves, flowers, and fruits of meadowsweet, which was also reflected in the high antioxidant and antibacterial activity of extracts from these parts. However, it should be noted that the roots and leaves of the lower part of the plant can also be considered valuable sources of groups of phenolic compounds such as catechins and tannins. Thus, the obtained results expand the data on the phytochemical composition and possible pharmacological use of meadowsweet plants, including through the study of previously unexplored parts of the meadowsweet, namely fruits, roots, and stems.

## Figures and Tables

**Table 1:** Content of some groups of phenolic compounds in the different parts of meadowsweet.

Plant Part	TPC ^1^, mg GAE g^−1^	TFC, mg RE g^−1^	THA, mg CAE g^−1^	TCC, mg CE g^−1^	PAs, mg CyE g^−1^	TTC, mg GAE g^−1^
Leaves (upper)	62.87 ± 1.86 a ^2^	117.42 ± 9.14 b	7.75 ± 0.52 a	12.15 ± 1.11 d	3.07 ± 0.23 a	27.59 ± 2.47 c
Leaves (middle)	59.62 ± 1.47 b	74.55 ± 5.36 c	3.64 ± 0.51 c	24.54 ± 2.00 c	1.73 ± 0.18 c	27.89 ± 2.27 c
Leaves (lower)	61.29 ± 1.85 ab	66.14 ± 4.60 c	4.21 ± 0.46 c	34.26 ± 2.67 b	2.32 ± 0.21 b	34.33 ± 1.72 ab
Stem (upper)	22.40 ± 0.47 d	6.15 ± 0.41 e	0.77 ± 0.06 d	1.57 ± 0.20 e	0.29 ± 0.03 d	8.28 ± 0.67 e
Stem (middle)	23.71 ± 1.05 d	2.11 ± 0.15 e	0.73 ± 0.02 d	2.83 ± 0.22 e	0.46 ± 0.05 d	11.53 ± 1.21 e
Stem (lower)	23.03 ± 0.69 d	1.54 ± 0.13 e	1.23 ± 0.17 d	4.53 ± 0.38 e	0.53 ± 0.06 d	17.80 ± 1.48 d
Flowers	64.65 ± 1.27 a	166.78 ± 7.68 a	8.68 ± 0.62 a	2.25 ± 0.09 e	0.55 ± 0.06 d	29.52 ± 2.60 bc
Fruits	63.66 ± 1.61 a	47.98 ± 4.46 d	6.39 ± 0.41 b	4.19 ± 0.26 e	0.67 ± 0.04 d	38.31 ± 2.50 a
Roots	53.12 ± 0.60 c	7.52 ± 0.44 e	1.43 ± 0.12 d	45.12 ± 3.02 a	3.37 ± 0.24 a	31.47 ± 2.21 bc

^1^ TPC—total phenolic content; TFC—total flavonoid content; THA—total hydroxycinnamic acid content; TCC—total catechin content; PAs—total proanthocyanidin content; TTC—total tannin content; GAE—gallic acid equivalents; RE—rutin equivalents; CAE—chlorogenic acid equivalents; CE—catechin equivalents; CyE—cyanidin equivalents. ^2^ Data were evaluated via one-way ANOVA followed by Tukey’s test. Different letters indicate significant differences among the means at *p* ≤ 0.05 (*n* = 4).

**Table 2:** Content of phenolic acids and flavonoids in the different parts of meadowsweet.

Compound	Retention Time, Min	Content of Individual Phenolic Compounds, mg g^−1^								
		Leaves (Upper)	Leaves (Middle)	Leaves (Lower)	Stem (Upper)	Stem (Middle)	Stem (Lower)	Flowers	Fruits	Roots
Flavonoids										
Catechin	9.7	1.98 ± 0.11	4.14 ± 0.28	6.28 ± 0.36	-	-	-	-	-	7.99 ± 0.61
Quercetin 3-*O*-rutinoside (rutin)	19.3	8.11 ± 0.46	4.76 ± 0.34	0.62 ± 0.06	0.16 ± 0.02	<LOQ ^1^	<LOQ	4.22 ± 0.30	2.06 ± 0.14	0.66 ± 0.04
Quercetin 3-β-d-glucoside (isoquercitrin)	19.9	5.34 ± 0.32	2.62 ± 0.18	2.28 ± 0.14	0.16 ± 0.02	0.074 ± 0.006	0.064 ± 0.004	2.40 ± 0.16	0.54 ± 0.04	0.048 ± 0.004
Quercetin 4′-*O*-glucoside (spiraeoside)	25.3	-	-	-	-	-	-	20.42 ± 1.42	2.36 ± 0.16	-
Luteolin 7-*O*-glucoside (cynaroside)	21.5	3.26 ± 0.16	<LOQ	<LOQ	0.10 ± 0.01	0.046 ± 0.006	0.12 ± 0.01	0.092 ± 0.004	0.32 ± 0.04	<LOQ
Kaempferol 3-*O*-glucoside (astragalin)	24.7	1.41 ± 0.08	-	0.12 ± 0.02	-	-	-	-	-	-
Phenolic acids										
Gallic acid	3.8	0.78 ± 0.08	0.78 ± 0.10	0.99 ± 0.06	1.02 ± 0.12	1.26 ± 0.10	0.72 ± 0.10	5.82 ± 0.40	4.32 ± 0.29	0.11 ± 0.01
3,4-Dihydroxybenzoic acid (protocatechuic acid)	5.8	<LOQ	0.028 ± 0.002	0.030 ± 0.004	0.024 ± 0.002	0.056 ± 0.004	0.082 ± 0.012	0.12 ± 0.01	0.092 ± 0.004	0.052 ± 0.004
Caftaric acid	9.2	1.12 ± 0.10	0.64 ± 0.04	0.56 ± 0.04	0.060 ± 0.004	0.052 ± 0.004	0.044 ± 0.002	2.92 ± 0.21	1.54 ± 0.09	0.070 ± 0.004
Chlorogenic acid	10.2	1.06 ± 0.12	1.31 ± 0.08	1.04 ± 0.06	0.11 ± 0.01	0.17 ± 0.01	0.19 ± 0.01	0.32 ± 0.04	0.58 ± 0.06	
Caffeic acid	10.5	-	-	-	-	-	-	0.14 ± 0.01	0.066 ± 0.007	-
*p*-Coumaric acid	14.2	0.44 ± 0.06	0.24 ± 0.04	0.17 ± 0.01	-	<LOQ	<LOQ	0.042 ± 0.007	0.72 ± 0.08	-
Ellagic acid	17.9	0.58 ± 0.04	0.38 ± 0.02	2.09 ± 0.12	0.18 ± 0.01	0.39 ± 0.03	0.48 ± 0.04	5.84 ± 0.34	3.44 ± 0.24	1.18 ± 0.08
Salicylic acid	19.1	-	-	-	0.98 ± 0.06	0.31 ± 0.03	0.19 ± 0.02	4.51 ± 0.32	1.94 ± 0.08	0.60 ± 0.04

^1^ LOQ—limit of quantitation.

**Table 3:** Antioxidant activity of the extracts from different parts of meadowsweet.

Plant Part	Antioxidant Activity, mg AsA g^−1^		
	DPPH	ABTS	FRAP
Leaves (upper)	172.3 ± 7.6 c ^1^	285.4 ± 12.0 c	130.9 ± 4.6 c
Leaves (middle)	118.8 ± 8.4 d	219.2 ± 8.5 d	111.7 ± 6.1 d
Leaves (lower)	127.8 ± 7.0 d	188.5 ± 5.8 de	136.6 ± 7.2 c
Stem (upper)	43.7 ± 4.1 f	75.7 ± 2.9 f	37.0 ± 3.3 e
Stem (middle)	45.4 ± 4.2 f	65.4 ± 4.1 f	32.1 ± 3.4 e
Stem (lower)	36.1 ± 3.1 f	76.1 ± 3.1 f	30.4 ± 2.2 e
Flowers	315.8 ± 13.6 a	415.7 ± 24.1 a	230.4 ± 7.1 a
Fruits	256.3 ± 9.5 b	367.8 ± 12.0 b	176.6 ± 11.0 b
Roots	82.0 ± 5.6 e	174.4 ± 6.3 e	125.3 ± 8.2 c

^1^ Data were evaluated via one-way ANOVA followed by Tukey’s test. Different letters indicate significant differences among the means at *p* ≤ 0.05 (*n* = 4). AsA—ascorbic acid equivalents.

**Table 4:** Antibacterial activity of meadowsweet extracts (the inhibition zones include the diameter of the disk—6 mm).

Plant Part	Inhibition Zone ^1^, mm							
	*Pseudomonas aeruginosa*				*Bacillus subtilis*			
	Extract Concentration, mg disk^−1^				Extract Concentration, mg disk^−1^			
	0.1	0.2	0.3	0.4	0.1	0.2	0.3	0.4
Leaves (upper)	7.2 ± 0.4	8.1 ± 0.3	10.7 ± 0.4	10.3 ± 0.6	–	–	–	–
Leaves (middle)	–	–	–	8.3 ± 0.4	–	–	–	–
Leaves (lower)	–	–	7.9 ± 0.5	8.2 ± 0.3	–	–	–	–
Stem (upper)	–	–	–	–	–	–	–	–
Stem (middle)	–	–	–	–	–	–	–	–
Stem (lower)	–	–	–	6.9 ± 0.3	–	–	–	–
Flowers	9.2 ± 0.1	10.7 ± 0.3	12.1 ± 0.6	13.3 ± 0.7	8.2 ± 0.2	10.3 ± 0.3	12.5 ± 0.2	13.1 ± 0.5
Fruits	9.6 ± 0.2	11.2 ± 0.4	13.4 ± 0.4	15.2 ± 0.6	–	–	9.7 ± 0.3	11.3 ± 0.5
Roots	–	–	9.2 ± 0.3	11.1 ± 0.3	–	–	–	–

^1^ Inhibition zones for the positive control (kanamycin, 25 µg per disk) were 18.4 ± 0.4 mm and 20.2 ± 0.6 mm in tests with *P. aeruginosa* and *B. subtilis*, respectively. For the negative control (10 µL of 8% DMSO per disk), no inhibition zone was observed.

## Data Availability

The data presented in this study are available on request from the corresponding author.

## Sample Availability

Samples of the plant material and tested extracts are available upon request from the authors.
